# Coupled echosounder and Doppler profiler measurements in the Strait of Gibraltar

**DOI:** 10.1038/s41598-024-82670-7

**Published:** 2024-12-28

**Authors:** Simone Sammartino, Jesús García-Lafuente, Irene Nadal, Ricardo F. Sánchez-Leal

**Affiliations:** 1 Physical Oceanography Group, University of Málaga, Málaga, Spain; 2 Instituto de Biotecnología y Desarrollo Azul (IBYDA), University of Málaga, Málaga, Spain; 3 Instituto de Ingeniería Oceánica (IIO), University of Málaga, Málaga, Spain; 4 Spanish Institute of Oceanography (IEO), Cádiz, Spain

**Keywords:** Strait of Gibraltar, ADCP, Velocity profile, Zooplankton diel vertical migration, Mediterranean outflow, Law of the wall, Ocean sciences, Physical oceanography

## Abstract

Long time series of velocity profiles collected by up-looking acoustic profilers in the westernmost sill of the Strait of Gibraltar show an unexpected pattern in the deepest ∼80 m of the water column, consisting in an appreciable diurnal weakening of the measured horizontal velocity. A harmonic analysis performed on long time series reveals a surprising magnitude of S_1_ constituent (exactly 1 cpd of frequency) in the horizontal velocity and echo amplitude, which prevails over the rest of diurnal constituents within this depth range, including K_1_, despite being around 200 times smaller than it in the tide generating potential. High resolution echograms collected by a new instrument recently installed in the mooring line, point at the diel vertical migration of living acoustic scatterers (zooplankton) as the most reasonable cause. It provokes a nightly depletion of scatterers availability near the bottom, which is registered by the instrument as a nighttime weakening of the velocity, as well as an increase of its uncertainty, at the deepest part of the profile. Newly acquired high spatial resolution measurements of the velocity near the seafloor report intense currents which are incompatible with the ones produced by the scatterers scarceness. This result indicates an overall underestimation of the Mediterranean current in previous works of approximately 17% within the depth range of 280–360 m, which in turn translates into an underestimation of previously computed outflow of ∼5%. These new findings make it necessary the re-computation of all the near-20-year long (to date) series of Mediterranean outflow based on the observations collected at this sill of the Strait of Gibraltar.

## Introduction

**Fig. 1:**
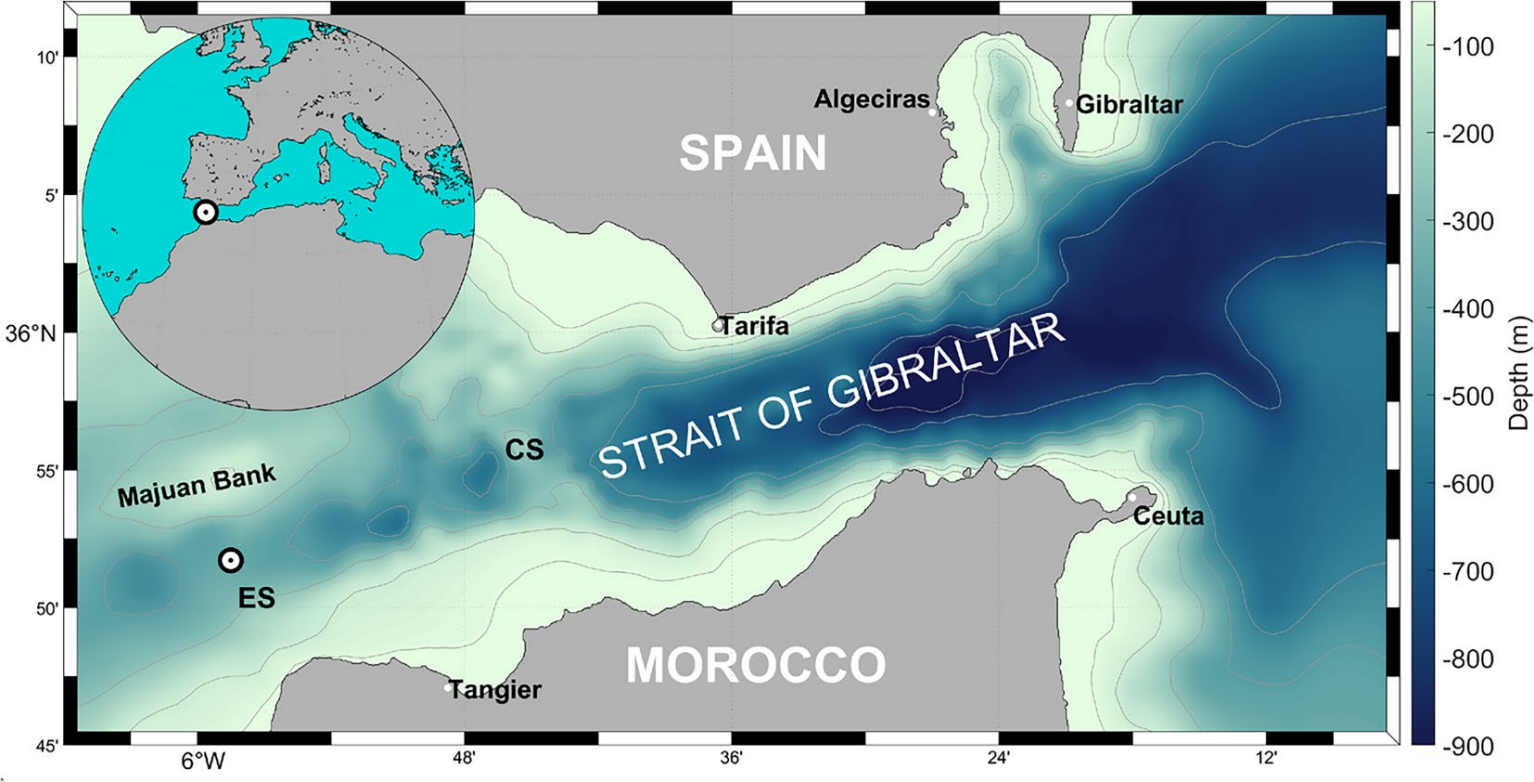
Map of the Strait of Gibraltar with the position (white circle) of the monitoring station in Espartel Sill (ES). The main Camarinal Sill is indicated by the acronym CS.

The semi-enclosed Mediterranean Sea suffers a permanent freshwater deficit due to the negative balance between precipitation, water runoff and evaporation^1^. The deficit is compensated by a net flow through the Strait of Gibraltar (SoG hereinafter, see Fig. 1), which is the result of a two-layer (baroclinic) exchange with the Atlantic ocean, where Atlantic water enters the Mediterranean basin at the surface and Mediterranean water flows out at depth^2–4^.

This basic state is periodically altered by a strong internal tide that arises from the interaction of a barotropic tide with the marked bathymetry of the SoG^5–10^. Subinertial fluctuations driven by changes of the atmospheric pressure field over the Mediterranean basin and by local winds, also modify the velocity structure of the water column^11–13^.

Accurate estimates of the exchanged flows through the SoG and their time variability is a necessary requisite of the oceanographic community studying the Mediterranean Sea. The issue has historically been approached by means of various strategies, including hydraulics^14,15^ and mass and energy balances of the Mediterranean basin^16^. In recent decades, numerical modeling used to simulate three-dimensional SoG dynamics has improved significantly our understanding of the link between Mediterranean circulation and SoG dynamics^17–24^. Both approaches are based on mathematical relationships between physical variables, which are solved either analytically or by numerical approximations, but that must be validated with observations. Therefore, in situ measurement become crucial, not only because they allow for making independent estimations by themselves, but also for their role in models validation. Observational campaigns are costly and spatially limited and the SoG is no exception. As the harsh environment it is, collecting observations there is a great challenge and building long reliable time series is a valuable achievement. For it, the measurements have to undergo rigorous quality control and be accompanied by a dependable measure of uncertainty.

The present study deals with the processing of the longest series of observed current velocities available in the SoG to date. The measurements were collected by a monitoring station deployed for the first time in 2004 in Espartel Sill (ES hereinafter, see Fig. 1), the last topographic constriction that Mediterranean waters encounter before sinking and spreading into the Atlantic Ocean. The location was chosen due to the stability of the Mediterranean outflow, which is rarely fully reversed here^25^, contrary to what happens in the main sill of Camarinal (CS) a few kilometers to the east (Fig. 1).

This paper revises recent works based on this dataset^26,27^ with emphasis on measurements collected by new instruments incorporated into the mooring line, which provide new observations that help to resolve questions still unanswered. The primary objective is the improvement of the accuracy of the velocity profiles with the aim of obtaining the best estimations of the exchanged flows and its temporal variability based on these observations. The paper is organized as follows: Section "A brief history of the monitoring station" describes the station and its evolution over the past 19 years. Section "Near-bottom velocity weakening" relates an unexpected behavior of the velocity profiles as the main core of the paper, while section "New insights on near-bottom dynamics" describes the advances provided by the acquired new data. Sections "Whole profile interpolation" addresses the post-processing of the vertical velocity profiles, and section "Summary and conclusions" draws some concluding remarks.

## A brief history of the monitoring station

**Fig. 2:**
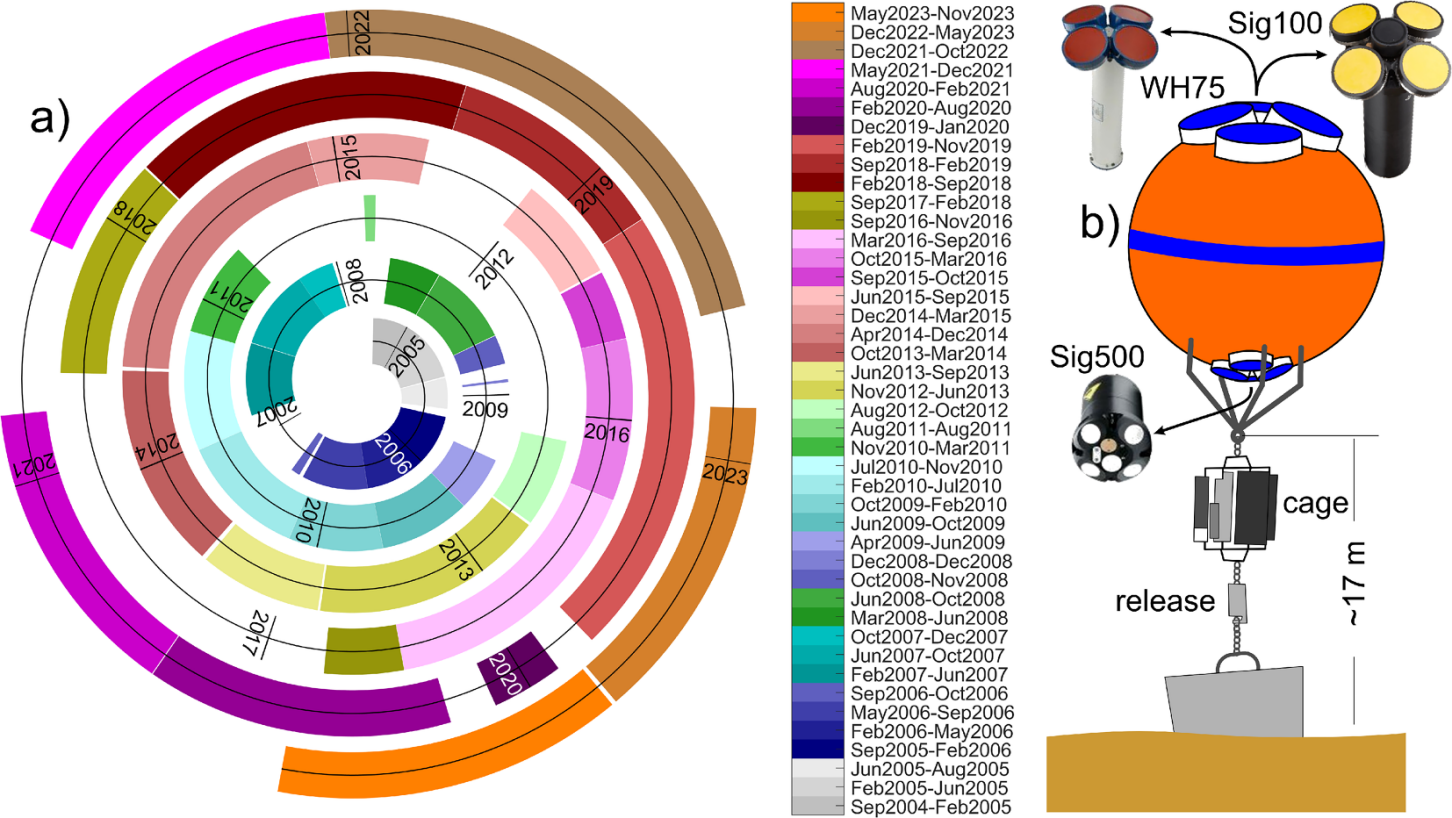
(**a**) Spiral plot of the 43 deployments in the ES from September 2004 to November 2023. The durations of the experiments are scaled over the radius of the spiral and they are not directly comparable. (**b**) Simplified sketch of the mooring structure. The buoy is not to scale with the rest of the structure.

In September 2004, the first 6-months deployment of the monitoring station at ES led off the longest series of current and thermohaline properties measurements ever collected in the SoG. The station is located in the southern channel of ES at a depth of approximately 360 m, and undergoes maintenance every 4–6 months. To date, 43 deployments have been completed (see Fig. 2a). The continuity of the series has been interrupted on few occasions, when the line suffered breaks or loss due to fishing activities or mechanical failure of the structure, which corresponds to 14.5% of the series length. Variables have been collected at regular sampling intervals of 30 min, although a higher temporal resolution is available since 2015 (see section ‘New instruments incorporation’).

The mooring line layout has undergone slight variations over time, but its main design has been maintained. It consists of a 1.5 m diameter subsurface buoy that is moored approximately 17 m above the seafloor. The buoy is anchored to a ∼1-ton concrete block through an acoustic release, which is operated to recover the line in order to collect data and maintenance tasks. A cage located 3 m below the buoy contains instruments that collect thermohaline and bio-geochemical water properties^27^.

### New instruments incorporation

The buoy was initially equipped with a single up-looking 75 kHz RDI Workhorse Long Ranger (WH75 hereinafter) Acoustic Doppler Current Profiler (ADCP hereinafter). In the last quarter of 2016, a Nortek Signature 500 ADCP (Sig500 hereinafter), with 500 kHz working frequency, configured in down-looking mode, was installed in the lower side of the buoy with the aim of investigating the so far unsampled bottom boundary layer (see Fig. 2b). In December 2019, a Nortek Signature 100 ADCP of 100 kHz (Sig100 hereinafter) was installed in a twin mooring line along with the WH75 for a test deployment, and in February 2020, it definitively replaced the WH75 in the main mooring line. As the WH75, the Sig100 has a four-beam Janus configuration with 20° slanted transducers. With a working frequency 33% higher than the WH75, it is capable to cover the whole water column with less blanking distance (2 m vs. 7 m), slightly better vertical resolution (5 m vs. 6 m bin thickness in our most recent configuration) and reduced sidelobe interference. The instrument features a more complex attitude measurement system than the WH75, known as the Attitude and Heading Reference System (AHRS), which utilizes a dedicated gyroscope to measure rotation, and is aimed at reducing the disturbances on tilt sensing caused by lateral movements^28^. Its most valuable advantage, however, is a fifth vertical beam that collects backscattered echoes at high spatial and temporal resolution. The echo system operates at a frequency range broader than the slanted beams (68–113 kHz). It transmits a linear chirp signal with a central frequency of 91 kHz and a bandwidth of 50%, resulting in five different bands of 73, 82, 90, 99, and 107 kHz with a bandwidth of approximately 9 kHz each. Echograms are collected at 20-s intervals and have a vertical resolution of 1 m.

One of the main differences of signature instruments with respect to the WH75, is that they can record both single-pings and ensemble averages, improving the recording capabilities of the RDI instrument. The availability of single-ping observations allows for the generation of varying sizes ensembles in the post-processing stage. This provides more accurate estimates of the ensemble uncertainty, computed as the ensemble standard deviation, with respect to the error velocity computed as the difference of the redundant vertical velocities, which are employed by RDI^29^ (see Appendix).

The vertical resolution of the up-looking profiler has improved over time, from an initial configuration of 8 m thick bins to its current 5 m. The down-looking ADCP has a bin thickness of 1 m.

## Near-bottom velocity weakening

**Fig. 3:**
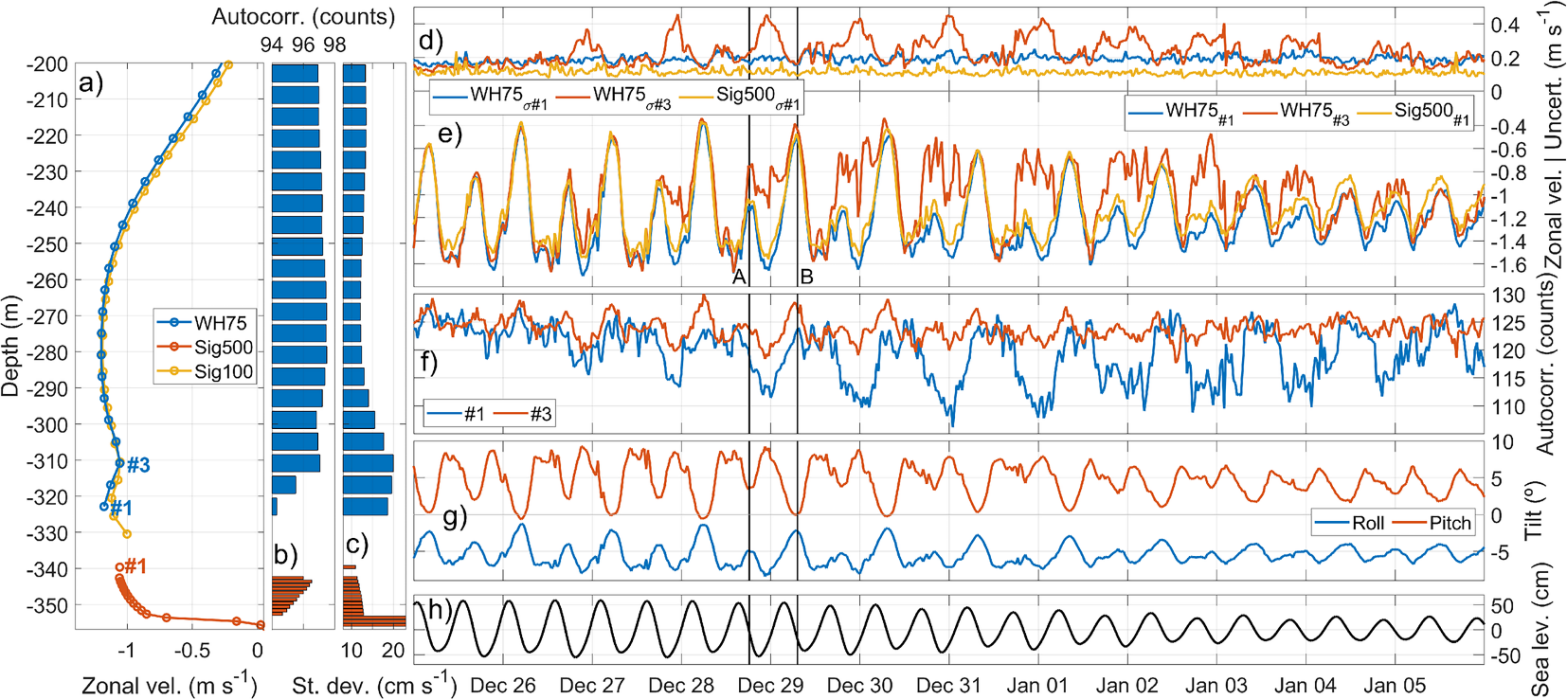
(**a**) Averaged zonal velocity measured with WH75 (blue), Sig100 (yellow) and Sig500 (red) ADCPs during the period December 2019–February 2020. Bins are numbered according to the instrument orientation: upwards for the up-looking WH75 and downwards for the down-looking Sig500. (**b**) Averaged autocorrelation of the backscattered echo. (**c**) Averaged zonal velocity ensemble standard deviation. (**e**) Time series of zonal velocity (negative values) and (**d**) its corresponding ensemble standard deviation of WH75 bins #1 and #3, and Sig500 bin #1 (see legend for color-coding) from 25th December 2019 to 6th January 2020. The two timestamps A and B, discussed in the text, are highlighted with labeled vertical lines. (**f**) Autocorrelation of WH75 bins #1 and #3. (**g**) WH75 roll and pitch. (**h**) Sea level measured by the Tarifa tide gauge (see location in Fig. 1).

Sammartino et al.^26^ discussed a near-bottom behavior of the current that was unexpected and difficult to explain. The time-averaged profile of the large time series analyzed showed a progressive decrease of the velocity from the depth of the westward maximum, downwards, coherent with the expected attenuation of the current strength toward the bottom. However, the deepest one or two bins depicted an reversal of that tendency and showed a sudden increase of the westward (negative) current (Fig. 3a, see also Fig. 3 in ^26^). The issue was addressed in ^27^, who investigated potential negative impacts of high instrumental tilts on the accuracy of the measurement without getting definitive conclusions. The inclusion of the two new instruments discussed above on the mooring line, allows for identifying new causes.

In December 2019, a short-term experiment with a specific configuration was carried out to simulate this feature with the WH75. We defined a 1 ping-per-ensemble sampling with a 6-s ping-rate to virtually obtain a single-ping series. The experiment allowed us to evaluate the effect of ensemble size on the measurement uncertainty and to confirm that the ensemble standard deviation is the most accurate estimation of the measurements dispersion (see Appendix for a detailed explanation of this subject). Figure 3a shows the deepest 155 m of the time-averaged vertical profiles of the horizontal zonal velocity (the major contributor to the total current), obtained with WH75 (blue symbols) and Sig500 (orange symbols). Figure 3b,c show the corresponding profiles of the averaged autocorrelation of the backscattered echo and ensemble standard deviation of the zonal velocity, respectively. Below the maximum westward velocity of the WH75 profile, located at approximately 280 m depth, both the autocorrelation and the standard deviation show a decrease in data accuracy. Specifically, the first two bins show a significant decrease in autocorrelation, which coincides with the anomaly of the velocity profile mentioned above.

Sig500 exhibits the expected logarithmic-shaped profile with a thin boundary layer and high velocities few meters above the bottom. The second and third bins were discarded due to inconsistencies caused by the shadowing of the instruments installed below the buoy (Fig. 2b). Autocorrelation decreases progressively with depth, and it is less than 90 counts in the three closest bins to the bottom (which falls below the lower limit of the x-axis in Fig. 3b). The standard deviation shows relatively constant values, except in these three bins, where it increases notably.

Figure 3e shows a portion of the zonal velocity time series of the first (WH75_#1_—blue line) and third (WH75_#3_—red line) bin of the WH75, and the first bin of the Sig500 (Sig500_#1_—yellow line). WH75 and Sig500 velocity series have been obtained as 300 and 50 pings-per-ensemble averages, respectively, with half-an-hour interval. They exhibit a distinct semidiurnal variability, with a significant diurnal inequality, modulated at subinertial scale. WH75_#1_ and Sig500_#1_ match most of the time (the three match at the beginning of the series), but WH75_#3_ shows noticeable weaker current every two semidiurnal cycles, particularly towards the end of the spring tidal cycle (see sea level in Fig. 3h). The discrepancy begins during the ebb phase of the tide, more specifically every two cycles of the ebb phase of the semidiurnal tide, when WH75_#1_ and Sig500_#1_ show much larger westward velocity than WH75_#3_ (e.g., instant A in Fig. 3e). The latter keep showing weaker current throughout an entire tidal cycle, but it matches again the values of the other two bins at instant B in the subsequent ebb tide when the current at WH75_#1_ and Sig500_#1_ is the weakest. Between these two ebb tides, the velocity in WH75_#3_ not only exhibits a clear discrepancy with WH75_#1_ but also higher fluctuations, as reflected by the periodic increase of the ensemble standard deviation (red line in Fig. 3d). The peaks can reach up to 60% of the relative uncertainty (standard deviation/velocity ratio). In contrast, the uncertainty in WH75_#1_ is relatively constant (around 15% of the corresponding velocity) and does not show periodicity. Autocorrelation patterns are the contrary (Fig. 3f): WH75_#1_ experiences clear daily drops that coincide with the maximum westward velocity every two cycles of the flood tide, whereas these drops are much more reduced in WH75_#3_.

In this energetic environment, the accuracy of measurements may be significantly limited by the tilt of the instrument^27^. Roll and pitch series shown in Fig. 3g do not exceed the manufacturer’s recommended maximum of 10°^29^, but it must be kept in mind that they are ensemble averages, and instantaneous tilts may be higher. Previous datasets exhibit tilt peaks up to 12° when the westward current was maximum. Excessive tilt may contribute to discrepancies in bottom layer measurements, although it is not the primary cause.

**Fig. 4:**
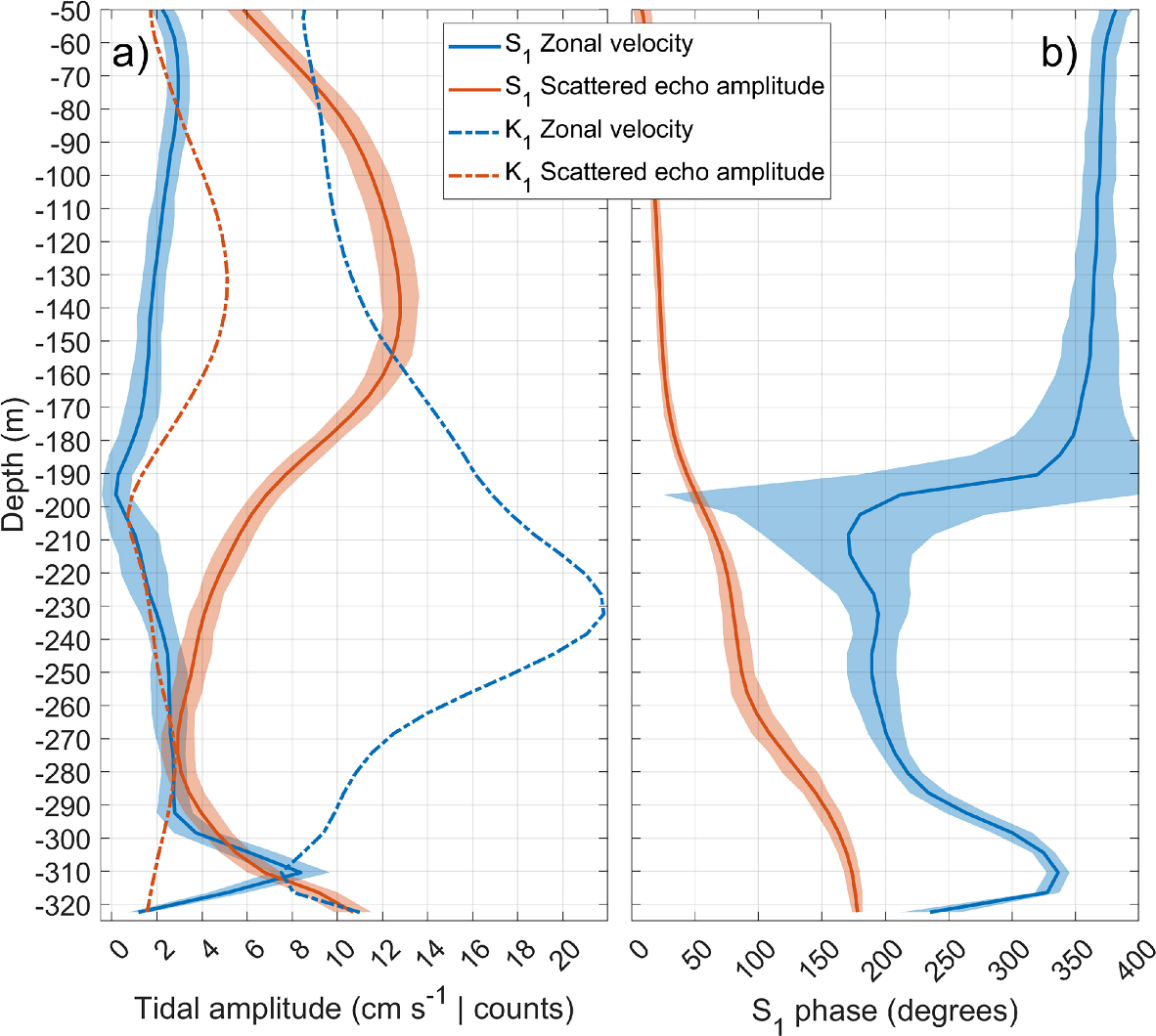
(**a**) Amplitude of S_1_ constituent computed on WH75 zonal velocity profile (blue lines) and scattered echo amplitude (averaged over the four beams in orange lines), during the period September 2018–December 2019. K_1_ amplitude profile of the zonal velocity (blue line) and scattered echo amplitude (orange line) are also shown in dot-dashed line. Zonal velocity and scattered echo are read as cm s^−1^ and dimensionless counts, respectively, in the same axes. (**b**) Same as (**a**) only for phase of the S_1_ constituent (degrees). In both panels, semitransparent areas depict ± 0.95 confidence intervals.

Sammartino et al.^26^ associated the periodic weakening of the deepest part of the velocity profile with a diurnal modulation of the tidal current. They analyzed the M_2_–K_1_ relation, but did not delve into its physical origin. A harmonic analysis^30^ of a 1.2-year-long series collected with WH75 between 2018 and 2019, which is long enough to resolve close tidal constituents according to Rayleigh criterion^31^, showed an unexpected relevance of S_1_ (exactly 1 cpd frequency) diurnal constituent at around 310 m depth, where its amplitude approaches 10 cm s^−1^ (Fig. 4a). This constituent is negligible in the tide-generating potential: its ratio with the prevailing diurnal constituent K_1_ (0.997 cpd) is around 1:200^32^. Thus, the expected S_1_ amplitude should be around 0.05 cm s^−1^ for a typical K_1_ amplitude of 10 cm s^−1^ (Fig. 4a). It must be said that S_1_ is often identified, as a small amplitude component, in long sea level time series, although its origin is not astronomic. It is associated with the radiational tide, a concept introduced by ^33^ to account for tidal oscillations caused directly or indirectly by the sun radiation, such as land-sea breezes or the daily atmospheric pressure oscillations transferred to the ocean through the barometric effect^34^. Obviously, these processes cannot be behind the relevance of S_1_ revealed by the harmonic analysis of the series illustrated here.

Figure 4a shows that although S_1_ current amplitudes are generally lower than 4 cm s^−1^, they peak to reach K_1_ values around 310 m depth, coinciding with the local minimum in zonal velocity depicted in Fig. 3a. The phase undergoes a rapid variation around 190 m, the mean depth of the interface^26^, and exhibits an out-of-phase pattern between the upper Atlantic and the lower Mediterranean layer. However, downwards from the depth of maximum average westward velocity (around 280 m, Fig. 3a) it begins to increase until reaching a maximum at the depth of the peak of S_1_ amplitude (Fig. 4b). At this depth, S_1_ is almost in phase with the Atlantic layer (a difference of only 30 degrees or 2 h), which is consistent with the weakening of the averaged profile at those depths shown in Fig. 3a. Within this depth range, the amplitude and phase of the scattered echo (orange lines) shows a progressive increase downwards. An average throughout the entire profile gives a S_1_ echo-amplitude almost three times greater than K_1_ (7.89 vs. 2.76 counts, compare solid orange line with dot-dashed orange line in Fig. 4a). At the depth of the S_1_ zonal velocity amplitude peak the echo oscillates with an amplitude of approximately 7 counts, in nearly phase opposition with the current: the weaker the westward current (the less negative) the lower the scattered echo strength. This suggests that the daily weakening of the Mediterranean outflowing velocity around 310 m depth illustrated in Fig. 3a may be caused by a decrease in echo amplitude.

Although the Sig100 ping rate has to be reduced with respect to the WH75 configuration due to higher battery consumption, the autocorrelation and velocity standard deviation of both instruments are fully comparable, with the former providing very similar profiles (see Sig100 and WH75 mean profiles in Fig. 3a, as an example). In fact, the unexpected weakening of the velocity discussed earlier is even more clearly illustrated by the higher resolution of Sig100 and its better response to severe tilt conditions (see Fig. 3a). The echograms registered by the instrument provide a new tool that helps to gain understanding on the phenomenon.

## New insights on near-bottom dynamics

**Fig. 5:**
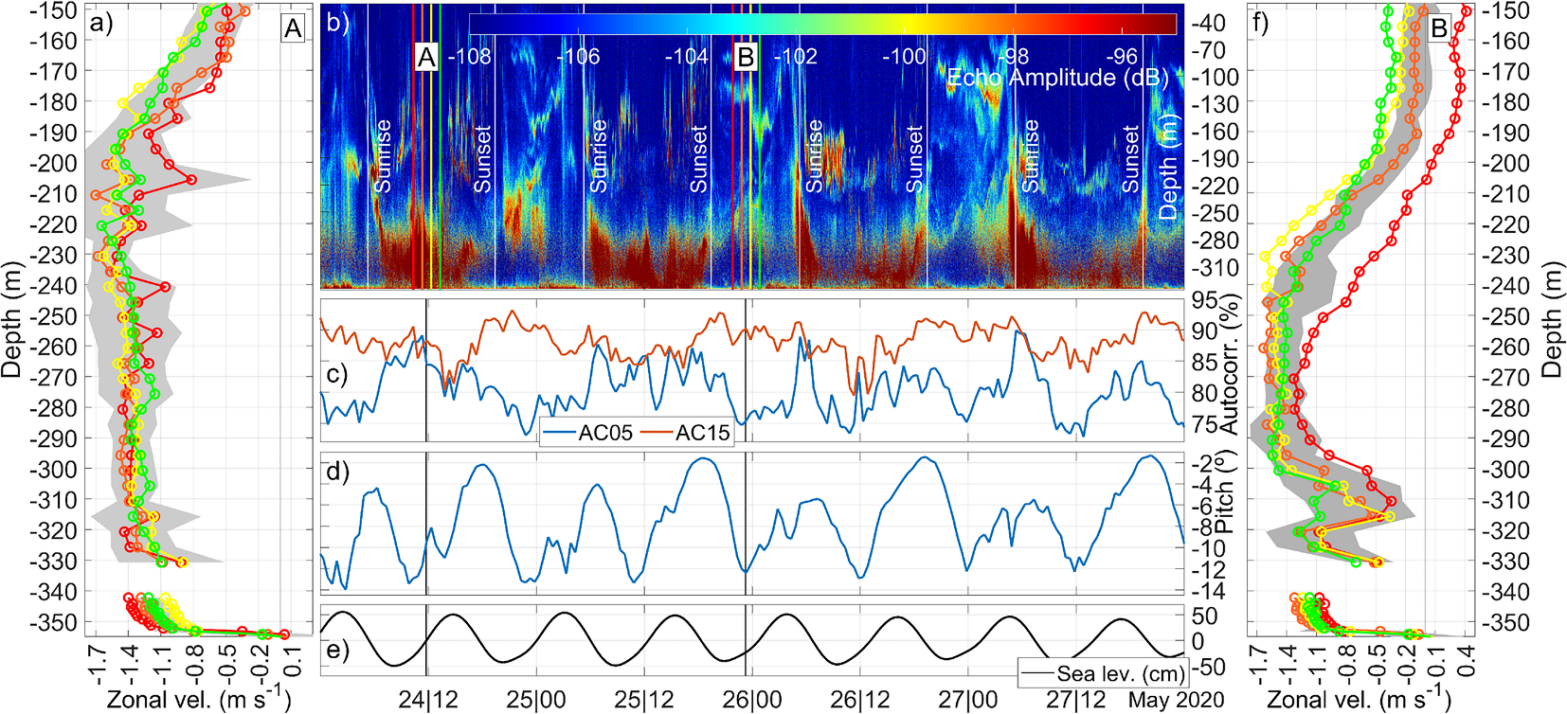
(**a**) and (**f**) Four zonal velocity profiles collected by Sig100 and Sig500 ADCPs around the time of maximum flood (3 h after low water, see (**e**) on 24th May, 2020 around 12:00 (set A) and on 26th May, 2020 around 0:00 (set B), respectively. Gray shadows represent the Sig100|Sig500 mean zonal velocity uncertainty (see text). (**b**) 99 kHz echogram (dB) collected with 2 0 s sampling interval and 1 m vertical resolution, between 24 and 28th of May, 2020. Vertical lines indicate the sunset and sunrise times at the mooring latitude. The time of the profiles displayed in panels (**a**) and (**f**) are indicated with the same color code. (**c**) Autocorrelation (%) of the backscattered echo averaged over bins 1–10 (AC05—from 285 to 330 m depth) and bins 11–20 (AC15—from 235 to 280 m depth). (**d**) Sig100 pitch (degrees). (**e**) Sea level measured in Tarifa.

Figure 5 shows a fragment of the dataset collected by the up-looking Sig100 and the down-looking Sig500 ADCPs between February and August 2020. Figure 5a,f display the deeper 200 m portion of four selected velocity profiles at the times labeled A and B in Fig. 5b, respectively, collected around three hours after low water in Tarifa (Fig. 5e), when maximal flood (westward) current is expected^7^. Accordingly, both sets of profiles exhibit strong westward velocity below the zone of maximum vertical shear, occasionally exceeding 1.5 m s^−1^. However, the pattern differs markedly in the lowest portion of the Sig100 profiles: profiles A (Fig. 5a) are rather constant and fit well with the profiles of the underlying Sig500, whereas profiles B (Fig. 5f) show a noticeable weakening of the current around 310 m depth (in the same manner as the averaged WH75 profile in Fig. 3a) and fit poorly with the Sig500 data. As a result, a thicker and more uniform westward-moving layer (∼140 m thick) is obtained at time A than at time B. Profiles A also show less dispersion, computed as the mean profile ± one mean standard deviation of the four profiles considered (gray shadow in Fig. 5a), particularly in the deeper 40 m layer. In contrast, profiles B are more scattered and the dispersion in those deeper bins increases significantly with the current weakening. The instrument tilt does not seem to be the issue in these cases, as the pitch has similar (although very high) values in both situations (Fig. 5d). Roll is nearly constant throughout the experiment and is not considered. The fact that Sig100 works properly with pitch peaks exceeding − 12° indicates a satisfactory performance of the instrument even under severe attitude conditions.

A primary distinction between the flood tides A and B is the time of occurrence: A is noon, B is midnight. The echogram in Fig. 5b reveals a clear and sudden decrease of echo amplitude below 270 m approximately during nighttime. Plankton is one of the most common reflectors employed in Doppler technology to measure current speed^28,29^. In particular, zooplankton^35^ and mesopelagic community^36^ are typically strong reflectors, while phytoplankton is almost transparent to the acoustic ping of ADCPs^37^. They undergoes Diel Vertical Migration (DVM) as a means of feeding on phytoplankton in the photic zone at night and avoiding visual predation during the day^38^. This migration matches the periodicity observed in the echogram, suggesting that the diurnal fluctuations of living sound scatterers are the cause of the dissimilitude of the profiles of Fig. 5a, f. Bottom echo amplitude patches clearly show upwards (downwards) displacements at sunset (sunrise), confirmed by corresponding positive (negative) values of the vertical velocity (not shown).

Similar deductions were obtained by ^37^ and ^39^. Acoustic transects over ES during the 1986 Gibraltar Experiment showed strong reflector accumulations not deeper than 200 m in nighttime, both in flood and ebb tide (Figs. 6.3a,b and 7.1 in ^2^), along with hints of downward migration of scatterers at dawn (Fig. 10.2 in ^2^). Using a 75 kHz up-looking ADCP to analyze plankton and internal waves interaction in the Alboran Sea (east side of the SoG), van Haren^40^ founds a dominant diurnal periodicity in the backscatter signal due to DVM, which was more intense during daytime when zooplankton grazed near the interfacial layers. Ursella et al.^35^ analyzed a 10-years time series of a 300 kHz and a 75 kHz ADCP moored in the Gulf of Mexico, and recognized both 24 h and 12 h periodicity in the vertical migration of different zooplankton taxa and micronekton. In our observations, the scatterers availability in the deepest portion of the profile in nighttime (e.g.: B series) appear to be much less than in daytime (e.g.: A series). The difference between the echo intensities at the bottom, as a proxy of the availability of zooplankton, is approximately 10 dB, which corresponds to a drop of approximately 1 order of magnitude in terms of power of the acoustic backscattered signal.

The negative effect of DVM on measurements accuracy is confirmed by the autocorrelation (Fig. 5c), strongly related to scatterers availability. The series of averaged autocorrelation of the 10 deepest bins of the Sig100 (AC05 series) exhibits clear periodic drops coincidental with the nighttime depletion of scatterers, while the averaged autocorrelation of Sig100 bins #11 to #20 (AC15 series) centered in a more uniform portion of the water column, is generally more stable. Both series show a good correlation with the echo amplitude, 0.69 ± 0.01 (0.01 being the 95% confidence interval) in the case of AC05 series and 0.50 ± 0.02 for AC15. In contrast, reflectors availability does not affect apparently the Sig500 profiles, possibly due to its five-times higher acoustic frequency and, perhaps, to some sediment resuspension expected near the bottom.

Figure 5 still hides few caveats, though. Echo amplitudes above ∼200 m are generally comparable to the low values observed in the deeper layers, which are being cited as the most probable cause of the velocity weakening. Why should the upper part of the water column provide significant velocity measurements with nearly the same level of acoustic response as the deeper layers, which in turn show high uncertainty? The crux of the matter lies in the way the ADCP operates.

**Fig. 6:**
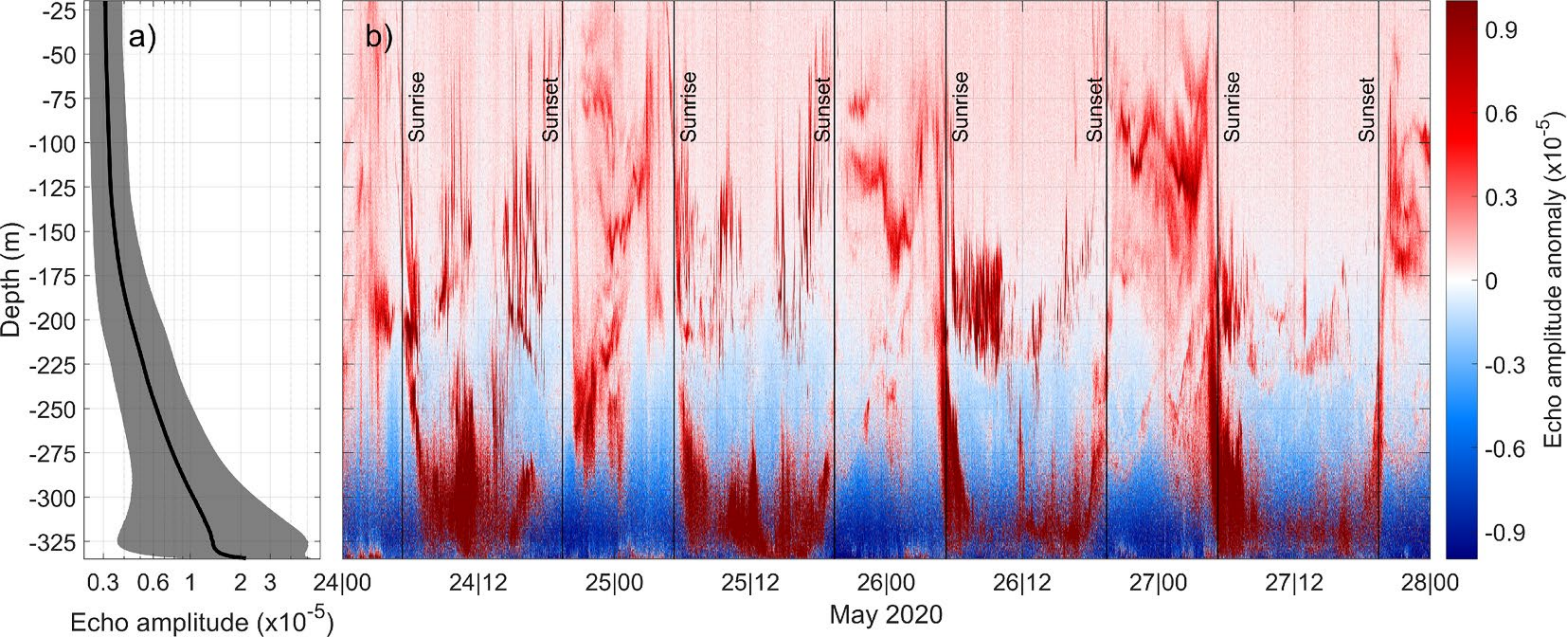
(**a**) Time average of the dimensionless-transformed 99 kHz echogram (quantity I - black thick line) with the associated standard deviation (gray area), computed for the whole experiment mentioned in the text. (**b**) Echogram anomaly obtained as the echo record subtracted by the average profile of (**a**). The time interval is the same of Fig. 5. The original echogram series has been backward converted to linear dimensionless quantities by using equation (2).

Acoustic signals emitted by the instrument attenuate by water absorption and beam spreading^29^, which results in a progressive loss of backscattered echo amplitude as we move away from transducer. The result is a progressive loss of backscattered echo amplitude with increasing distance from the transducers. The recorded scattered echo amplitude, known as the Signal-to-Noise Ratio (formally defined as the strength of the acoustic signal relative to the background noise level^28^), is given by:

1 $$SNR = 20\log_{10} \left( {\frac{{A_{s} }}{{A_{n} }}} \right)\, \left[ {{\text{dB}}} \right]$$

where *A*_*s*_ and *A*_*n*_ are the physical amplitudes of the received signal and a reference (the floor noise), respectively. This quantity is the one directly provided by the instrument. The dimensionless quantity *I*:

2 $$I = \frac{{A_{s} }}{{A_{n} }} = 10^{{\left( {\frac{SNR}{{20}}} \right)}}$$

has been calculated here to illustrate the upward attenuation of the echo (Fig. 6). In particular, Fig. 6a depicts the vertical profile of the quantity *I* averaged throughout the whole experiment duration. It clearly shows how the echo amplitude decreases with distance to the transducer, as prescribed by the SONAR equation^28^. Figure 6b depicts the echogram anomalies, obtained by subtracting the averaged profile of Fig. 6a from the whole *I* series, a procedure not applicable to the SNR directly because of its logarithmic dependence. Notice that either Fig. 6a profile and Fig. 6b series are dimensionless units and not dB, as in the corresponding echogram series of Fig. 5b. The result is a sort of usable echo amplitude, which is the backscattered echo weighted by the level of the transmitted signal reaching a certain distance from the instrument.

Above ∼200 m depth, the anomalies are always positive, indicating sufficient availability of scatterers throughout the upper part of the water column. Negative anomalies are observed below this depth, with the strongest minima concentrated at the depth of the velocity weakening (see also Figs. 3a and 5f) and only at nighttime. This leads to a remarkable inflation of the standard deviation of the deepest bins of the averaged profile in Fig. 6a. Here the zooplankton DVM appears even clearer, with patent rises (descents) of the maximum positive anomalies at sunset (sunrise), and higher concentrations (positive anomalies) of reflectors distributed throughout the upper part of the water column at night. In addition, the echogram anomaly shows significant high frequency fluctuations of the echo amplitude maxima in the upper part of the water column during the day.

**Fig. 7:**
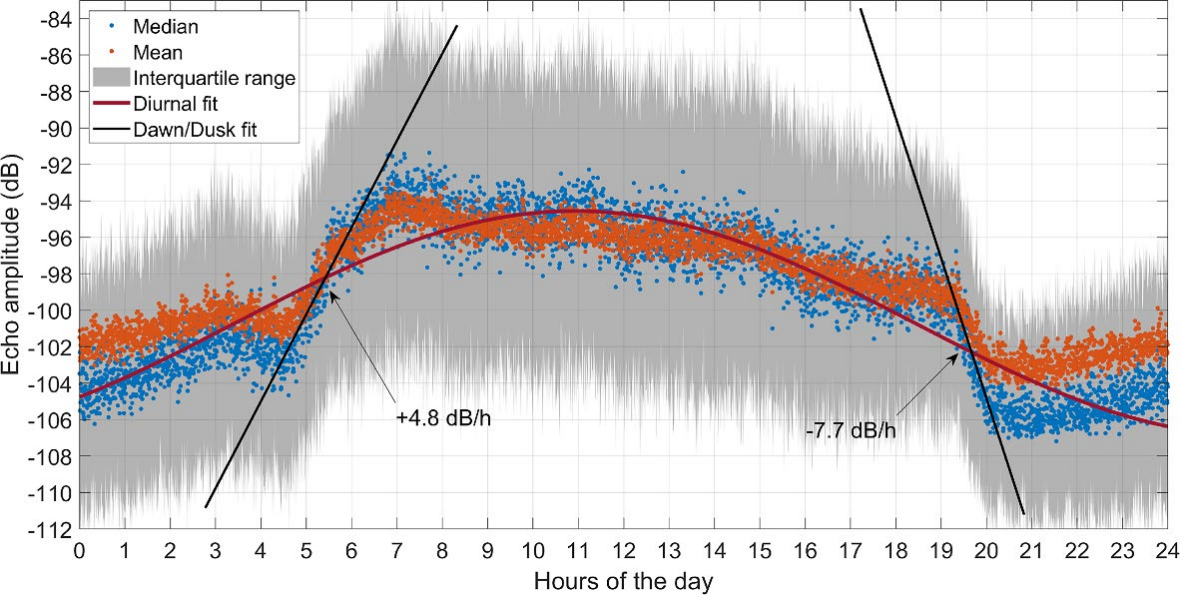
Median (blue dots) and mean (orange dots) of the echogram time series extracted at 310 m depth grouped every 20 s over an entire day. Gray area depicts the interquartile range centered in the median values. Purple line is the 1 cpd harmonic fit and the two black lines are the linear fits of the portion of the series around dawn (left) and dusk (right), with indication of the linear slope.

The relationship between echogram intensity and the DVM is furtherly illustrated in Fig. 7. It shows the diurnal cycle of the echo amplitude (dB in this case) of the bin located at 310 m, the depth of the velocity weakening (Fig. 3a), obtained as median (blue dots) and mean (orange dots) of all the records grouped every 20 s (the echogram sampling rate) throughout the 24 h of the day. Lower amplitudes are observed from dusk to dawn (nighttime) when living reflectors move upwards, and higher amplitudes during daytime when they move downwards. Once more, it strongly suggests that the well-depicted echogram diurnal cycle is due to the zooplankton DVM. Within the interquartile range of variability (gray shaded area), a difference between median and mean values are observed in nighttime, indicating an asymmetric (positively skewed) distribution of the samples. This means that higher amplitudes are less likely to occur than other values, suggesting a long-term modulation of the phenomenon whose origin can be ascribed to either the spring-neap tidal cycle^40^, or to the phytoplankton spring bloom^41^, which secondarily drives zooplankton vertical dynamics. Although the series length is insufficient for obtaining robust long-term analysis, Fig. S1 in supplementary material online confirms this hypothesis. The same analysis performed in Fig. 5 for late March shows a more stable response of the echograms to the higher scatterers availability during spring bloom.

Despite its diurnal periodicity, the circadian fluctuations are far from being harmonic (see harmonic fit—purple line—in Fig. 7). Zooplankton rises and sinks at faster rates than prescribed by the sinusoidal fit, with downward motions at dawn being slower (+ 4.8 dB/h) than the upward ones at dusk (− 7.7 dB/h). Velasco et al.^37^ observe similar behavior in the Mediterranean Sea. The difference appears to be related to the grazing activity of the organism. The greater the vertical speed at the beginning of the food search during sunset, the more efficient the grazing, while sinking rate becomes more passive during the downward vertical migration at sunrise^42^.

The instantaneous velocity profiles typically display a degree of spikiness associated with the strength of the current in question. In general, profiles with higher velocities exhibit greater spikes, particularly in the vicinity of the maximum depth of the Mediterranean westward current. The irregularity of the instantaneous profile can be assessed by comparing it with the logarithm-like vanishing profile expected in bottom layer dynamics (see section "Whole profile interpolation"). The Root Mean Square (*R*) series of differences between the measured profile and the theoretical fit, is compared with the instrument tilt (*T*), strictly associated to the line drag, which in turn is related to the tidal strength (spring-neap tide) cycle. The correlation coefficient is 0.66 ± 0.01, indicating a clear relation between instrument tilt and profile spikiness. The correlation coefficient between *R* and the averaged echo amplitude of the first 50 bins of the echogram (*E*) is lower: 0.39 ± 0.02. However, excluding extreme tilt events (pitch > 12°), the correlation coefficient between *R* and *T*, and between *R* and *E* series, decreases to 0.59 ± 0.01 and increases to 0.48 ± 0.02, respectively. Instrument tilt seems to affect the accuracy of the entire profile, distorting it when the pitch exceeds that plausible critical threshold, while scatterers availability systematically alters the deeper portion of the profile to the extent that even the time-averaged velocity profiles depart from their expected pattern (Fig. 3a). The high-frequency variability of the current, and turbulence may also be considered as potential explanations for this current measurement bias. However, this would not account for the diurnal periodicity of such behavior. On average, the noon current maximum is even stronger than the nighttime one (see, for instance, Fig. 5). If turbulent energy dissipation is accepted as the cause of the current bias, it should act in both maxima, and even more markedly in the noon one.

The hypothesis that the velocity measured by the ADCP is sensitive to the abundance of reflectors in the sense that the smaller the number of reflectors, the lower the reliability of the recorded velocity, would explain the anomaly depicted in Fig. 3a. And, more specifically, the unexpected importance of S_1_ constituent, whose frequency matches exactly the diurnal cycle and, therefore, makes it to be the most disturbed. As far as this pattern is consequence of the DVM, the velocity profiles must be revised and recalculated to avoid this spurious effect.

## Whole profile interpolation

**Fig. 8:**
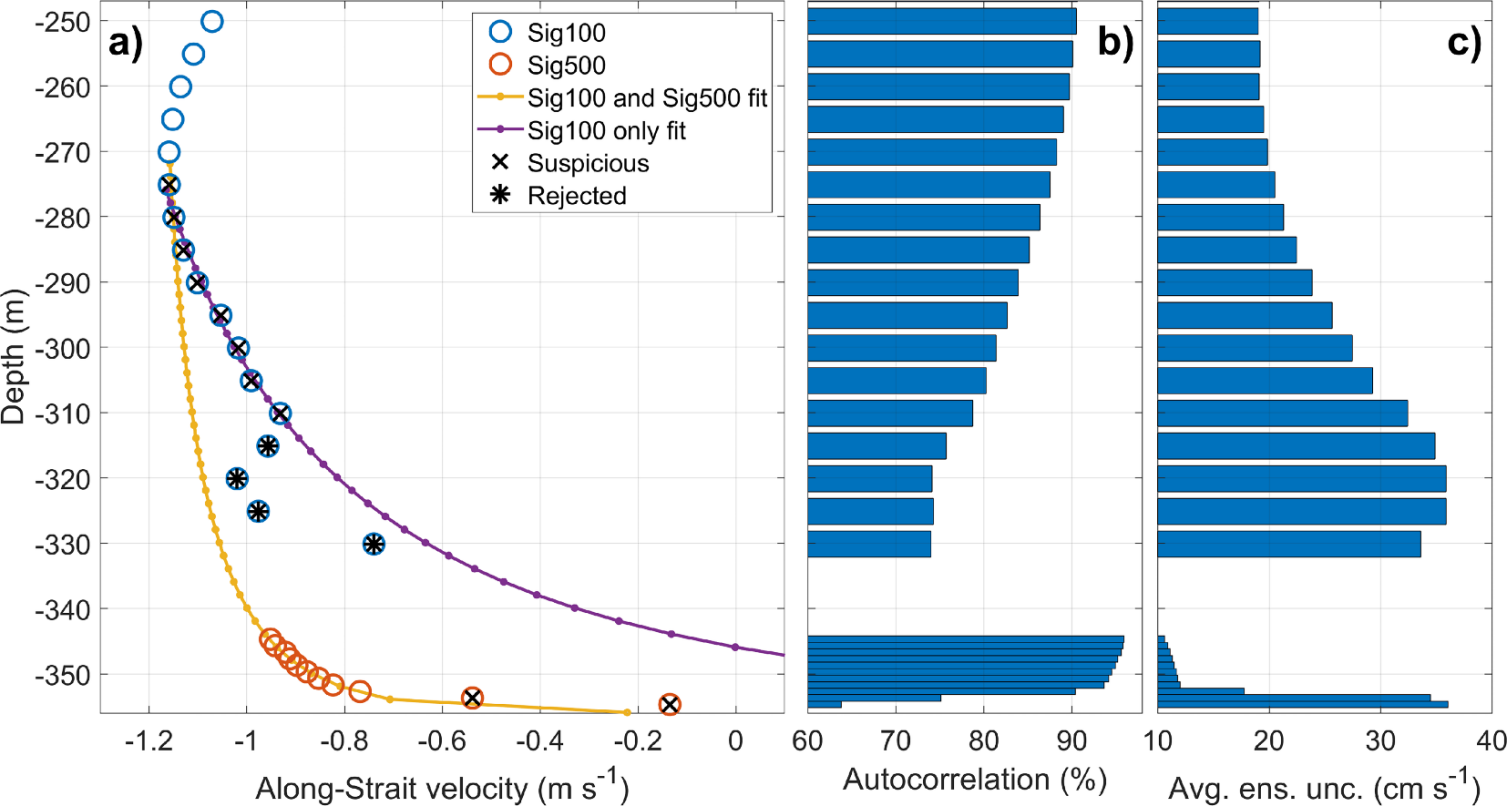
(**a**) Deepest 100 m of the time-averaged profile of along-strait velocity (May to December 2021). Sig100 and Sig500 velocity data are depicted in blue and red circles, respectively. Rejected and suspicious bins are marked with asterisks and crosses, respectively. Yellow line is the fit to Sig100 and Sig500 data after removing rejected and suspicious bins. Purple line is the fit to Sig100 data only, after excluding the rejected bins. (**b**) Averaged profiles of autocorrelation. (**c**) Averaged profiles of standard deviation of the ensembles (averaged ensemble uncertainty).

Electronic limitations of instruments and their sampling configuration give rise to a systematic gap of approximately 20 m between the lowermost bin of the up-looking profile of WH75 and the uppermost bin of the down-looking profile of Sig500 ADCPs. The gap reduces to 15 m when the Sig100 replaces the WH75 (Figs. 3a, 8a). To compute the Mediterranean outflow the gap must be filled, for which a similar approach to that discussed in ^27^ for coupling WH75 and Sig500 profiles is followed here but using the Sig100-Sig500 pair in this case along with the outcome of the echogram analysis.

Figure 8a shows the deepest 100 m of Sig100 and Sig500 profile of along-strait velocity (velocity is rotated 17° anticlockwise—see ^26^) averaged from May to December 2021. Figure 8b,c show the autocorrelation and the averaged ensemble uncertainty. Both metrics indicate a decrease in the reliability of Sig100 data below the depth of maximum westward velocity, which coincides with the region most affected by the scatterers fluctuations mentioned earlier. With this in mind, all the bins below that maximum are labeled as suspicious (cross-coded) and the four deepest bins characterized by the lowest autocorrelation and maximum uncertainty are rejected (asterisks-coded). Notice that these bins exhibit a clear deviation from the expected vanishing behavior of the rest of profile. Sig500 bins exhibit higher autocorrelation than Sig100 bins, except for the two deepest bins, which have significantly higher uncertainty due to the sidelobe interference with the seafloor. Therefore, these bins are labeled as suspicious too.

The data gap has been filled by interpolating the profiles with a modified version of the widespread law of the wall^43^. It prescribes the current speed near the seafloor as:

3 $$u\left( z \right) = \frac{{u^{*} }}{k}\ln \left( {\frac{{z\left( {h - z_{0} } \right)}}{{z_{0} \left( {h - z} \right)}}} \right)$$

where u* is the friction velocity, related to the shear stress at the bottom, *z*_0_ is the height above the bottom where velocity is assumed to be zero (known as roughness length^44^), and *k* is the dimensionless von Kármán constant, empirically estimated as 0.41. The length scale *h*, strictly related to the Ozmidov scale^43^, provides one degree of freedom more than the standard law of the wall^44^, thus improving the fit of the observed data^27^ and obtaining slightly better performance than in ^26^.

Equation (3) was least-squares fitted to the Sig100 and Sig500 averaged profiles, with the suspicious and rejected bins removed (Fig. 8). This includes the bin of maximum westward velocity measured by the Sig100 and the valid bins of the Sig500. The fit provides an interpolated profile (yellow line in Fig. 8a) with reasonable friction velocities of few cm s^−1^ and length scales of few tenths of cm. These values match well with estimations for the Mediterranean outflow plume in ^45^ and ^43^. Purple line in Fig. 8a shows the outcome of fitting Eq. (3) exclusively to Sig100 bins, with only the rejected bins filtered out. The fit produces an unreliable bottom profile, with an estimated friction velocity and length scale of approximately 35 cm s^−1^ and 10 m, respectively. This result highlights the significance of the Sig500 measurements in accurately characterizing the bottom layer dynamics, as anticipated by ^26^, who were unable to use them at that time. While a direct comparison between the two interpolated profiles of Fig. 8a may not be entirely fair nor significant, it is still noteworthy that the Sig500 measurements indicate significantly higher velocity near the bottom than those extrapolated by only the up-looking ADCP. Even the linear extrapolation to the seafloor of the deepest Sig100 data (including the suspicious and rejected bins and assuming no-slip conditions at the seafloor) provides interpolated near-bottom velocities that are considerably underestimated compared to Sig500 observations (results not shown). The maximum averaged difference exceeds 50 cm s^−1^ over the deepest 30 m.

The good agreement of the fitted yellow curve and averaged velocity profile depicted in Fig. 8a is also achieved with the instantaneous profiles. Therefore, the aforementioned approach is applied throughout the entire series. At each time step the maximum (negative) current is included as the only one valid bin of the Sig100 to be least-squares fitted together with the Sig500 measurements (with the two deepest bins removed). In addition to Eq. (3), a simpler linear model is also fitted, and, if the *r*^2^ value of the former is less than 0.9, the latter is used as the interpolating function. On average, the 70% of the profiles are interpolated using the modified law of the wall. It is important to mention that the bins below the depth of maximum westward velocity, which are systematically excluded from the fit, generally match the interpolated profile. However, when the scattered echo intensity decreases, the velocity in these bins departs from the profile and the fit provides a more reliable profile of the near-bottom current.

This approach has also been applied to the older WH75-Sig500 series. Prior to the down-looking ADCP installation (see Fig. 2 for reference) the gap beneath the first valid bin of the up-looking ADCP has been filled using a single virtual measurement located 50 cm above the seafloor with a fixed current speed of − 3 cm s^−1^. This value is the average of the first non-zero velocity at the bottom measured by the Sig500 ADCP during its 7-year working life.

## Summary and conclusions

A local velocity minimum in the deeper portion of the averaged velocity profile collected by a low-frequency up-looking ADCP moored in the westernmost exit of the SoG during 19 years has been investigated in detail. The expected downwards vanishing profile of the westward Mediterranean current is periodically altered, exhibiting unreasonably weak velocities few tents of meters above the bottom.

The recent replacement of the ADCP with a new one, equipped with an additional fifth beam capable to collect high-resolution echograms of the water column, allowed to shed light into this anomaly. A long-term harmonic analysis indicates an unusual relevance of S_1_ constituent not only in the profile of velocity but also in the scattered echo, suggesting that some kind of non-physical process is acting with diurnal periodicity. The daily fluctuation of the availability of living scatterers, related to zooplankton diel vertical migrations, is the alleged explanation of the velocity weakening, and the most reasonable strategy chosen to overcome this failure involves the removal of the deepest bins of the up-looking ADCP.

The integration of a second down-looking, higher frequency, ADCP supported this hypothesis and provided new high spatial-resolution data in the bottom layer. These data appear to be nearly unaffected by the fluctuations of scatterers and reveal the existence of high current speeds near the bottom (periodically exceeding 80 cm s^−1^ just 5 m above the seafloor), corroborating the spuriousness of the anomaly observed by the other ADCP. The recalculated profiles indicate an underestimation of the deepest ∼80 m of the Mediterranean velocity computed so far^26,27^ by approximately a 17%. This corresponds to an underestimation of the Mediterranean outflow, whose calculations are not detailed here, of approximately the 5%.

## Electronic supplementary material

Below is the link to the electronic supplementary material.

Supplementary Information.

## Figures and Tables

**Fig. A1:**
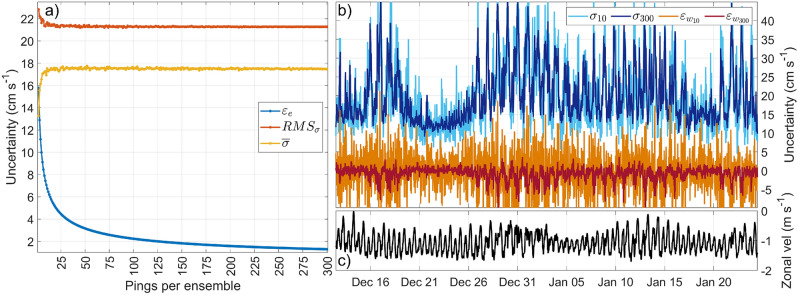
(**a**) Ensemble uncertainty (blue line), and average and RMS (yellow and red line, respectively) of the zonal current standard deviation of the ensembles obtained from 2 to 300 ppe in the period December 2019–January 2020. The ensemble uncertainty is computed as $\varepsilon_{e} = \varepsilon_{p} /\sqrt N$, with *N* the varying ppe, and *ε*_*p*_ = 22.3 cm s^−1^ the single ping standard deviation as prescribed by ^29^. (**b**) Zonal current standard deviation of ensembles obtained by averaging 10 (light blue) and 300 (dark blue) pings, velocity error averaged over 10 (light orange) and 300 (dark red) pings. (**c**) Zonal current during the measured period. All quantities are computed at the third bin from the bottom, corresponding to the maximum averaged uncertainty (see Fig. 3c).

## Data Availability

The datasets analyzed during the current study are available in the OceanSites repository, https://www.ocean-ops.org/board. Please access freely the board and search for “Espartel Sill” site. A direct link has not been provided by the database manager.

## Appendix: Single ping measurements and ensemble uncertainty

The WH75 standard configuration applied up to 2015 prescribed ensemble intervals of 30 min and a total amount of 50 pings per ensemble, with a ping-to-ping interval of 36 s. As usual, single pings were used internally to provide averaged ensemble measurements, and then discarded. This setup guarantees very reliable measurements and provides accurate outflow estimations (e.g.: ^26^), but it does not allow to directly assess ensemble uncertainty. Generally, ADCP current measurement uncertainty is addressed in a dual manner. The first one is the ensemble uncertainty defined as $\varepsilon_{e} = \varepsilon_{p} /\sqrt N$, where *ε*_*p*_ is the single ping standard deviation, and *N* is the amount of pings averaged in the ensemble^29^. The single ping uncertainty depends on internal factors such as the transmit pulse length, the bin thickness and the beam geometry^28^. The ensemble averaging reduces this uncertainty proportionally to the number of pings involved. Since it depends on the very configuration of the instrument, the ensemble uncertainty may only be considered as a nominal background noise, which cannot take into account the effect of high frequency current fluctuations, or instrument motion. The second metric used to estimate current uncertainty is the difference between the redundant measurements of the vertical velocity, available in a four-beam ADCP configuration. This is considered as a measure of the validity of the assumption of homogeneous velocity field^46^, and may be intended as more of a proxy of the current uncertainty than a direct estimation of it. It is referred as velocity error and will be denoted as *ε*_*w*_.

In this framework, single pings availability may allow more robust estimations of the current measurements uncertainty, and its dependence on the own current strength. The shortness of the mooring deployed in December 2019–February 2020 (see Fig. 3) allowed to configure the WH75 with a 6 s ping-to-ping interval, corresponding to a half-an-hour ensemble of 300 pings. With this, we simulated different ensembles sizes during the post-processing of current data: for each 30 min set of current samples, a series of fictitious ensembles have been computed with a number of pings per ensemble (ppe hereinafter) varying from 2 to 300, and, for each ensemble, the averaged current and its standard deviation (*σ*) have been computed. Notice that the fictitious pings have been evenly distributed throughout the ensemble duration.

Figure 9a shows the median (yellow line) and the Root Mean Square (RMS, red line) of the standard deviation of the zonal current of the third bin, computed for all the ensembles sizes from 2 to 300 ppe. The corresponding ensemble uncertainty *ε*_*e*_ (blue line) has been computed following as explained above, with *ε*_*p*_ = 22.3 cm s^−1^, as prescribed by ^29^ for the WH75 instrument. Unexpectedly the standard deviation of sampled current behaves oppositely to the ensemble uncertainty, while it is the RMS curve that shows a decreasing asymptotic behavior like the *ε*_*e*_ curve. This result suggests that *ε*_*e*_ resembles the ensemble uncertainty dispersion (RMS) more than the uncertainty itself. However, although they behave similarly, remarkable differences arise. The *ε*_*e*_ curve shows the typical shape of a deterministic function dropping as *N*^1/2^, while the RMS curve presents a much earlier flattening curve, nearly specular to *σ* curve. It initially increases because of the inclusion of newer and newer pings to the ensemble with increasing ppe, and then shortly tends to a constant value asymptotically.

By fitting a damping exponential equation $\sigma = \sigma_{0} e^{ - BN} + C$ to the *σ* curve (with *σ*_0_ the initial standard deviation, *B* the damping coefficient, *N* the ppe and *C* the asymptotic ceil of the curve), we obtain an asymptotic uncertainty of 17.5 cm s^−1^, reached at 15 ppe, computed as five times *B*, the *e*-folding value. The standard deviation computed so far represents a reliable and more realistic measure of the current uncertainty, which in fact stabilizes at a certain size of the ensemble, while the *ε*_*e*_ curve only offers a theoretical reference of the nominal instrument accuracy. Notice that the value of the RMS curve at ppe = 1 is 22.5 cm s^−1^, nearly coinciding with the single ping uncertainty, 22.3 cm s^−1^ prescribed by ^29^, somehow confirming the correspondence between *ε*_*e*_ and RMS, more than *ε*_*e*_ and *σ*.

The ensemble standard deviation reflects more fairly the effect of the own current intensity and its temporal variability. Figure A1b shows the time series of the current standard deviation computed for 10 (light blue) and 300 (dark blue) ppe. In both curves a clear subinertial modulation is appreciable, with maximum uncertainty around the maximum current strength under spring tide (see Fig. A1c). Standard deviation is lower (less *σ*, 16.9 cm s^−1^ vs. 17.5 cm s^−1^) and slightly more scattered (more RMS, 21.5 cm s^−1^ vs. 21.3 cm s^−1^) in the 10 ppe series than the 300 ppe, as anticipated by Fig. A1a. Time series of the velocity error *ε*_*w*_, computed as difference of the redundant vertical currents, are displayed in orange and red, for 10 and 300 ppe, respectively. They resemble a white noise signal, with nearly null temporal variability and unreliable averaged absolute values of 0.7 cm s^−1^ and 0.8 cm s^−1^, respectively. The corresponding ensemble uncertainty is 7.1 cm s^−1^ and 1.3 cm s^−1^, respectively, again not a reliable estimation of the observed variability of Fig. A1b.

Notice that the ensemble standard deviation series of Fig. A1b reflect a right-skewed distribution of the values (skewness > 1 for both series), with many low values and less periodical higher peaks, thus supporting the use of the median, more than the mean, to describe their centrality. All these calculations have been made for the zonal current of the third bin from the bottom which presents the maximal dispersion (see Fig. 3c), but they are equally valid, although shifted toward lower uncertainties, for the other bins.
