# Supplementary material for: Coupled echosounder and Doppler profiler measurements in the Strait of Gibraltar
Source: Sci Rep. 2024 Dec 28;14:31261. doi: 10.1038/s41598-024-82670-7 (PMC11682202; doi:10.1038/s41598-024-82670-7)
Supplement: Supplementary file 1 — Supplementary Information. [file 41598_2024_82670_MOESM1_ESM.docx]

COUPLED ECHOSOUNDER AND DOPPLER PROFILER MEASUREMENTS IN THE STRAIT OF GIBRALTAR

Simone Sammartino, Jesús García-Lafuente, Irene Nadal, Ricardo F. Sánchez-Leal

SUPPLEMENTARY MATERIAL

Figure S1


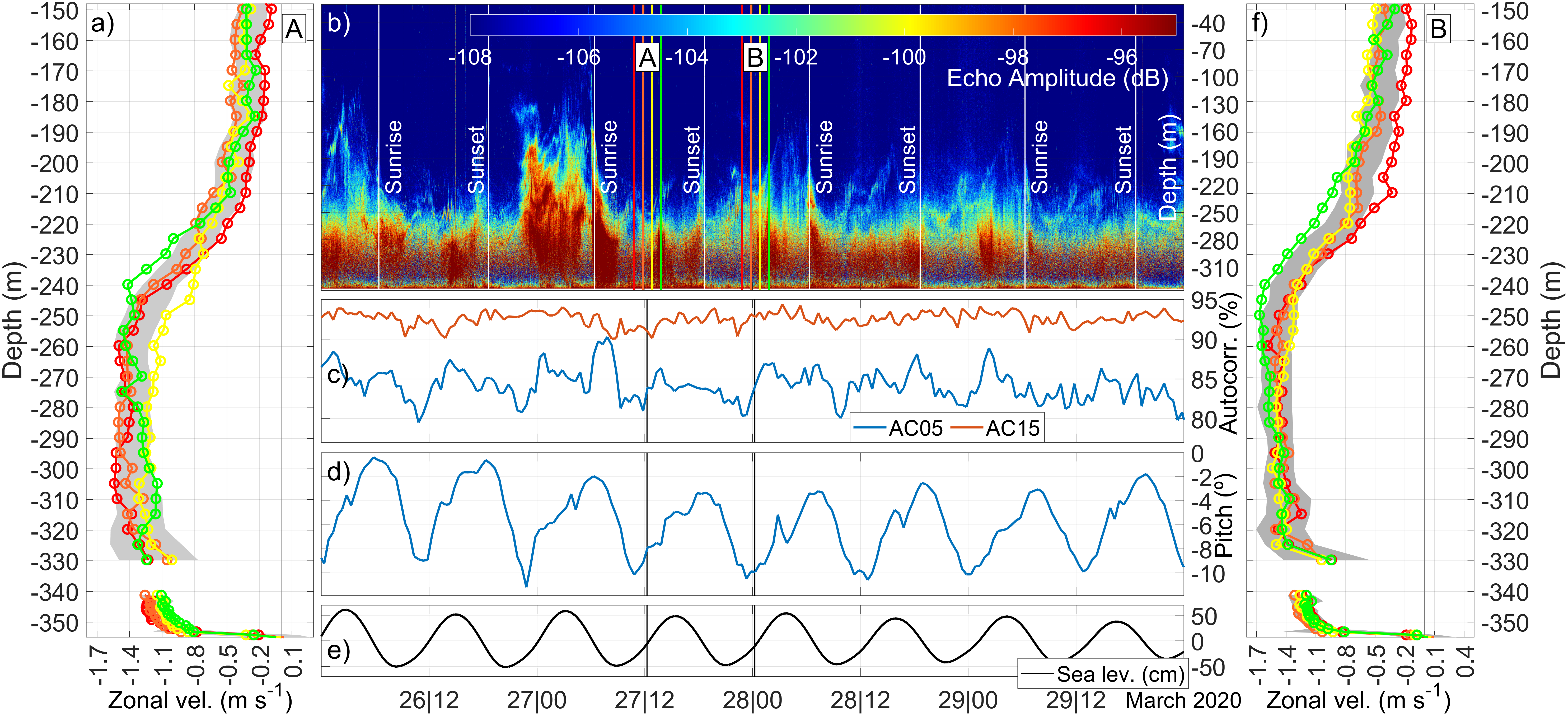


*Figure S1 – As in Figure 5 for the period 26^th^ – 30^th^ of March 2020.*
